# Feasibility of aquaculture cultivation of elkhorn sea moss (*Kappaphycus alvarezii*) in a horizontal long line in the Tropical Eastern Pacific

**DOI:** 10.1038/s41598-023-41795-x

**Published:** 2023-09-07

**Authors:** Milton Montúfar-Romero, Raúl E. Rincones-León, Lorena Belén Cáceres-Farias, María Mercedes Espinoza-Vera, Ulises Avendaño, Teodoro Cruz-Jaime, Luis Cubillos, Walter Ruiz, Willan Revelo, César Lodeiros, Alonzo Alfaro-Núñez, Lenin Cáceres-Farias

**Affiliations:** 1 Programa Acuicultura, Instituto Público de Investigación de Acuicultura y Pesca, Proceso Investigación de los Recursos Bioacuáticos y su Ambiente, Guayaquil, Ecuador; 2 Programa de Doctorado en Ciencias con mención en Manejo de Recursos Acuáticos Renovables, Facultad de Ciencias Naturales y Oceanográficas, Universidad de Concepción, Concepción, Chile; 3 Agromarina Biorma Aquaculture C.A., Porlamar, Venezuela; 4 AquaCEAL Corporation, Urb. Las Palmeras, Ave. Capitán Byron Palacios & General Quisquis, #8, EC230101 Santo Domingo de los Colorados, Ecuador; 5 Programa de Maestría en Administración de Empresas, mención en Gestión de Procesos Organizacionales, Universidad Tecnológica Equinoccial, Vía Chone Km. 4 1/2 y Av. Italia, 17.24.23.1 Santo Domingo de los Colorados, Ecuador; 6 Grupo de Investigación en Biología y Cultivo de Moluscos, Departamento de Acuicultura, Pesca y Recursos Naturales Renovables, Facultad de Acuicultura y Ciencias del Mar, Universidad Técnica de Manabí, Calle Gonzalo Loor Velasco, EC131459 Bahía de Caráquez, Ecuador; 7 Cooperativa de Producción Pesquera Artesanal Santa Rosa de Salinas, Santa Elena, Ecuador; 8 Centro COPAS Coastal, Departamento de Oceanografía, Facultad de Ciencias Naturales y Oceanográficas, Universidad de Concepción, Concepción, Chile; 9 Department of Clinical Biochemistry, Naestved Hospital, Ringstedgade 57a, 4700 Naestved, Denmark; 10 Section for Evolutionary Genomics, GLOBE Institute, University of Copenhagen, Øster Farimagsgade 5, 1353 Copenhagen K, Denmark; 11 Maestría de Investigación en Acuicultura, Instituto de Posgrado, Universidad Técnica de Manabí, Bahía de Caráquez, Manabí Ecuador

**Keywords:** Agroecology, Environmental economics

## Abstract

Seaweed aquaculture has become a profitable and an attractive alternative of cultivation thanks to its quick biomass production for food, feed, and other non-food applications. In addition, the ecosystem services generated by seaweed cultivation towards carbon fixation represents a more sustainable solution to the ocean’s acidification. The growth of elkhorn sea moss (*Kappaphycus alvarezii*) was evaluated in three plots with 200 propagules during a period of 70 days in a floating raft system covered by a fishing net underneath. Initial weight of propagules was 159.3 ± 12.74 g in wet biomass and 15.3 ± 1.43 g in dry biomass and were sampled up to 19 days (in the lag growth phase; period I), up to 33 days (in the exponential growth phase; period II) and up to 70 days (in the stationarity growth phase; period III). The variations of sea surface water temperature, salinity, turbidity (Secchi depth), total ammonium, nitrites, nitrates, and phosphate were determined. The growth increase was more evident in the exponential phase II when a dry biomass of 28.0 ± 2.48 (1153.3 ± 6.25 g in wet mass) was reached, more than 7 times the biomass of propagules with an average daily growth rate of 15.2% g.day^–1^. The carrying capacity of the zone was estimated at 86.2% in the area where 53 cultivation units would be projected. The economic analysis presented a financial feasibility with a net profit of 19% over the projected income and an IRR of 16.5%, recovering the investment in an estimated period of 4.3 years. We recommend to continue with larger-scale studies to optimize the cultivation of *K. alvarezii* in the study area.

## Introduction

In 2022, more than 35 million tons of wet macroalgae were produced worldwide, generating a turnover of approximately 1.9 billion dollars^1^. In addition to their economic importance, algae production plays an important role in the ecological balance of aquatic ecosystems, contributing to the reduction of CO_2_ and eutrophication^2^. Therefore, they are an important tool in climate change mitigation, as algae culture can promote the elevation of the pH of water in their aquaculture areas, thus combating the acidification of the water^3^.

One of the species with the highest production in the tropics is the alga cottoni or elkhorn sea moss *Kappaphycus alvarezii* (Doty) Doty ex Silva, a species of red algae mainly exploited to produce carrageenan, commonly used as a food additive, but also in the pharmaceutical industry^4^. Moreover, recent studies propose the cultivation of *K. alvarezii* bioproducts for other uses in the nutraceutical field^5^.

The elkhorn sea moss *K. alvarezii* grows naturally in areas of Southeast Asia, mainly in Indonesia, Malaysia, and the Philippines at depths between 1-17 m^6^. It usually grows in warm waters (27–30 °C), with salinities between 30 and 35 ‰^7–9^, under high light levels^10^ and an intense degree of water movement^11^. In addition, the growth of *K. alvarezii* does not require water with a high nutrient content for its development^11–14^ and has a relatively faster growth rate than other macroalgae’s species^15^. Moreover, during the last decades the cultivation of the *K. alvarezii* has also been expanded to further circumtropical latitudes throughout the world, including Fiji, the Philippines, Malaysia, Tuvalu, the Maldives^16^, India, Tanzania^17^, Vietnam, Cambodia, and Myanmar^16,18^. In addition, cultivation of *K. alvarezii* has also successfully been implemented in Latin America in countries with tropical climates such as Brazil^19^, Cuba^14^, Venezuela^20^, Mexico^4^, Belize, Lesser Antilles^21^ and Colombia^21,22^, thanks to the inherent advantages previously mentioned of fast and easy production that this species has in comparison to other endemic.

In Ecuador, the cultivation of the elkhorn *K. alvarezii* began in 2014 with the government initiative through the Aquaculture Undersecretariat in association with artisanal fishermen (Santa Rosa de Salinas Artisanal Fishing Production Cooperative)^23,24^. Thus, *K. alvarezii* was included in the list of species suitable for mariculture as a “species under investigation” in 2017^25^ and considered as one of the promising species for the diversification of aquaculture in the country. While there are no published records yet of its aquaculture and economic feasibility, this represents one of the main goals in our study.

Considering that one of the most used seaweed farming systems are horizontal floating rafts^18,26^, in this study we examined it, within the framework of best harvest yield over time (up 19, 33 and 70 days). This is the first time, *K. alvarezii* has been growing in a system of floating rafts in the Tropical Eastern Pacific, and thus determining environmental factors associated with the modulation of its growth. Finally, productive, and socioeconomic projections of its cultivation in Bahía Las Conchas, province of Santa Elena, Ecuador, are proposed.

## Materials and methods

### Location and culture system

**Figure 1:**
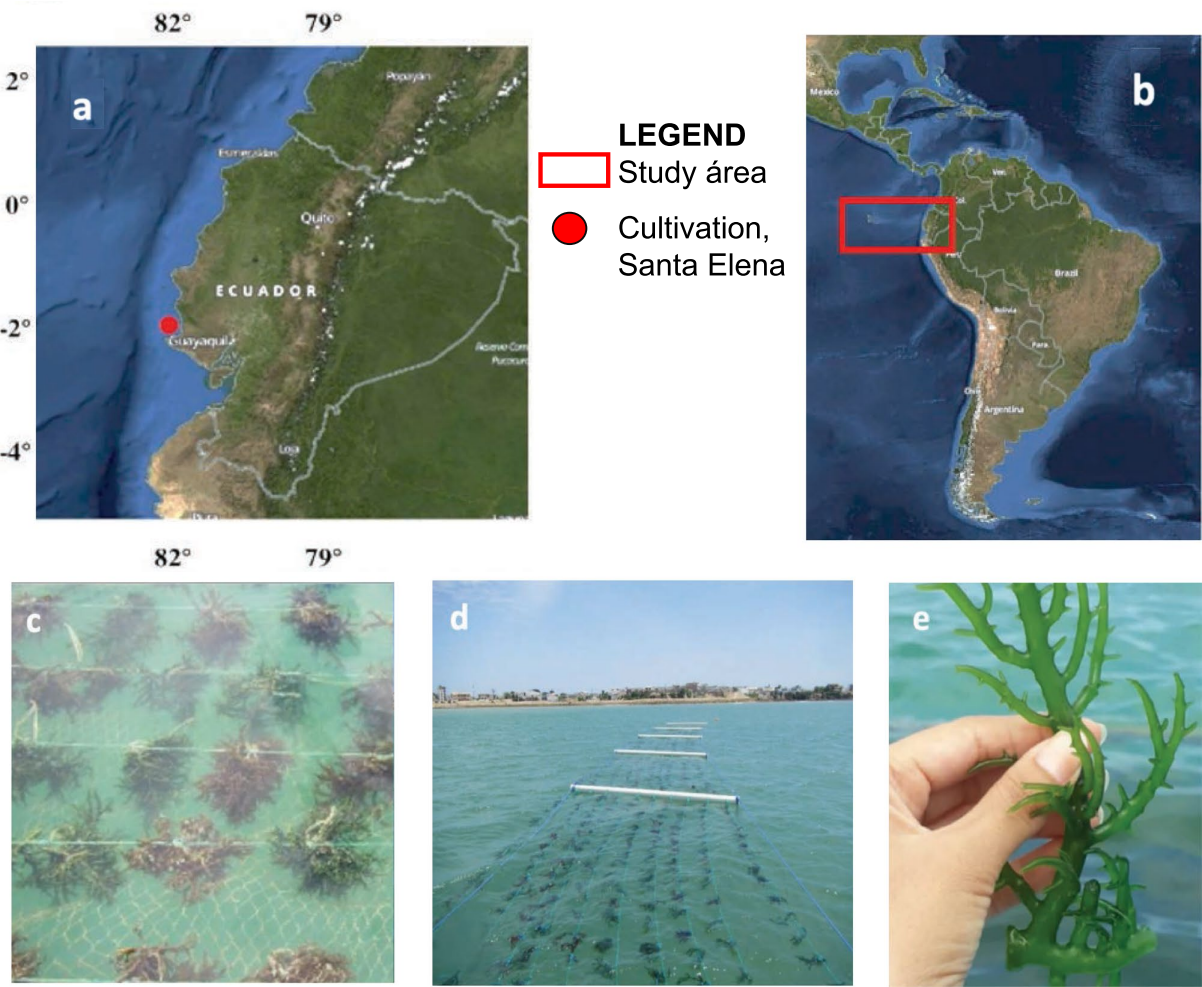
Panels (**a**) and (**b**) indicate the location of the cultivation site in the waters of Santa Elena province, Ecuador in South America. Panel (**c**) the propagules attached to the rope. Panel (**d**) a series of floating raft units, and finally panel (**e**) a branch of *Kappaphycus alvarezii* (Map source NASA: https://soto.podaac.earthdatacloud.nasa.gov/).

The study was carried out during a period of 3 months (July, August, and September in 2016) within the concession area of the Santa Rosa Artisanal Fishing Production Cooperative, located in Bahía Las Conchas, province of Santa Elena, Ecuador (Fig. 1). This area has a sandy substrate with depths of about 8–10 m^23^. The cultivation was carried out in a system of floating rafts using 110 mm diameter PVC tubes with a length of 3 m and 3 mm polypropylene ropes. These structures were fixed to the bottom by means of 250 kg cement weights located at an average depth of 5.4 ± 0.10 m. The cultivation unit was made up of 15 cells of 15 m^2^ (5 × 3 m) each for a total area of 225 m^2^. In each cell, 10 lines of 5 m length were placed, to which an average of 20 implants separated by 0.2 m each were attached. These cultivation structures were covered underneath with a fishing net to minimize the dispersion of detached seedlings and avoid possible herbivory by fish.

### Culture experiments

Within the floating rafts system, 3 non-contiguous experimental culture units (5 × 3 m) were chosen, each unit being considered as an experimental replica. In each experimental unit, 200 propagules were sown (manually fixed to the culture unit with a piece of polyester rope) with an initial weight of 159.3 ± 12.74 g of wet biomass and 15.3 ± 1.43 g of dry biomass (95% confidence interval as dispersion index in all measurements).

Three sampling periods were conducted after sowing at up to 19 days (in the lag growth phase; period I), at up to 33 days (in the exponential growth phase; period II) and at up to 70 days (in the stationarity growth phase; period III). For the calculations of wet biomass growth, a sample of 15 tissue samples was taken at random in each period, which were weighed in situ with a digital portable scale (0.01 g precision). From this sample, 3 tissue samples were randomly taken for the evaluation of dry biomass, which were washed with fresh water and dried in an oven at 105 °C for 3 h following the recommendations of Ohno et al.^7^. The rest of the tissue samples were returned to the respective experimental replica.

For the growth curves we used the absolute dry weight end in each of the cultivation periods. For comparative use of other *K. alvarezii* cultures we use the equation of the daily growth rate in percentage (% DGR) in wet mass, proposed by Yong^27^ with the Eq. (1) as follows:

1 $$\% \, DGR \, = [\left( {Wf - Wo} \right)^{1/t} - 1] \times \, 100,$$

where *Wo* is the initial weight (g), *Wt* is the final weight (g), and *t* is the number of days of culture. The data is presented as mean daily growth rates for periods I, II and III, respectively.

### Environmental factors

To estimate the possible influence of environmental factors on algal growth, sea surface water temperature and salinity were measured using a YSI Professional Plus (Pro Plus) multiparameter probe. Similarly, water samples were taken to determine the concentration of total ammonium (NH_4_^+^), nitrites (NO_2_^–^), nitrates (NO_3_^–^) and phosphate (PO_4_^3–^) using a HI 83,200 Hanna instruments® equipment, previously calibrated by colorimetric analysis (0.01 ppm of precision).

### Carrying capacity of culture area

As a feasibility factor in the study area, the carrying capacity was defined as the cultivation area that can be used for the mariculture activity of the macroalgae in a continuous way, considering that there are no social and ecological conflicts in the coastal system^2^. The calculations were based on the methodology applied by Azis^28^ taking into consideration the following Eqs. (2), (3) and (4). In this way, the system presented an area of 225 m^2^ (45 m length × 5 m width-crop unit) (*L*_*1*_*P*_*1*_). Additionally, a space between cultivation units of 10 m on all sides was considered, so the total projected area per cultivation unit was established at 1625 m^2^ (65 m length × 25 m width) (*L*_*2*_*P*_*2*_). The capacity of the water body was calculated according to the relationship:

2 $$Capacity \, of \, water \, body \, \left( \% \right) \, = L_{2} P_{2} {-} \, L_{1} P_{1} / \, L_{2} P_{2} \times \, 100\% ,$$

where *L*_*1*_ = Width of a culture unit,

*L*_*2*_ = Appropriate width of a culture unit,

*P*_*1*_ = Length of a culture unit,

*P*_*2*_ = Appropriate length of a culture unit.

The method used to calculate the adequate area without exceeding the load capacity of a specific area was based on the following relationship formulas:

3 $$Carrying \, capacity \, \left( {ha} \right) \, = \, Wa \, \times \, Wc,$$

where *Wa* = area (*ha*),

*Wc* = capacity of water body (%).

The maximum number of culture units that the carrying capacity of the water body can support was calculated using the following formula:

4 $$Number \, of \, culture \, units \, = C/A,$$

where *C* = Carrying capacity of the water body (*ha*),

*A* = maximum area of use.

### Economic viability

To determine the economic feasibility of cultivation of *K. alvarezii*, profitability was estimated with data on the maximum load capacity of the cultivation area, which was projected with a total of 53 floating rafts of 45 × 5 m of simple construction with floats of PVC pipes with a diameter of 110 mm and a length of 3 m. These rafts had a capacity of 150 lines or 2134 propagules. Seaweed production was estimated at the harvest time with the highest yield assuming 11 harvests/year (harvests of 30–35 days). The operational costs included an already operational infrastructure for maintaining the propagules as well as various activities (drying, packaging, salaries, mobility, etc.) necessary for the commercial production of macroalgae. Calc software functions were used to determine costs and financial indicators^29–31^. The financial analysis was projected over 10 years based on export prices for the weight of dehydrated seaweed as well as current local market prices for used inputs, expressed in US dollars. The projected revenue from the sale of the algae was based on a 4% annual increase in the sales price in the main international markets located in the Philippines, Indonesia, and Tanzania^32^.

### Statistical analysis

The growth rates of the dry biomass of *K. alvarezii* during the initial, middle, and final periods were compared using a one-way ANOVA, after verification of the normal distribution and homogeneity of variances in the treatments (Shapiro–Wilk and Levene’s tests, respectively), followed by Tukey’s post hoc tests, according to recommendations of Zar^33^. Data of the environmental factors were analysed using the non-parametric Kruskal–Wallis test, establishing differences between periods using paired comparisons of Dwass-Steel-Critchlow-Fligner, and following the recommendations of Hsu^34^. The significance level for all tests was set at *P* = 0.05.

### Ethics declaration

The seedlings of *K. alvarezii* were imported from Punta Laurel sector, Bocas del Toro Archipelago, in the Republic of Panama, with its respective phytosanitary certificate of origin and invoice. The experimental farm in Panama has been certified by the Undersecretary of Aquaculture as an exporting establishment of *Kappaphycus* macroalgae, being certified free of any pathological agent under the National Government of the Republic of Panama through the National Directorate of Animal Health of the Ministry of Agricultural Development.

## Results

### Growth

**Figure 2:**
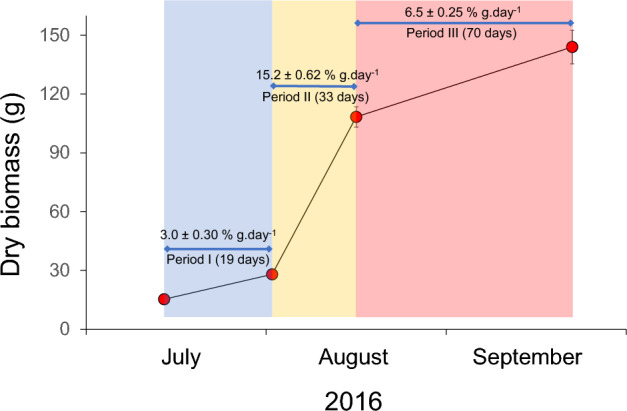
Growth estimates presented in absolute values of dry biomass of *Kappaphycus alvarezii* under cultivation in a floating rafts system on the coast of the province of Santa Elena, Ecuador. Numbers presented above the figure indicate mean % daily growth rates for periods I (from 0 to 19 days), II (from 19 to up 33 days) and III (from 33 to up 70 days). Vertical lines indicate 95% CI, confidence intervals. The three colours highlight the corresponding three different sampled periods from July to September 2016.

No mortality was observed, nor was there any evidence of damage or grazing on the *K. alvarezii* cultivation during the study period. The final wet biomass average was 1620.7 g ± 12.74 g (95% CI, confidence interval), representing more than 10 times the initial one (159.3 ± 12.74 g). The increase was more evident in the period II when a wet biomass of 1153.3 ± 6.25 g was recorded. The growth shown in absolute values of dry biomass was similar, reaching an average initial dry biomass of the propagules (15.3 ± 1.43 g) to 28.0 ± 2.48, 108.3 ± 5.17 and 144.0 ± 8.61 g, for the I period (up to 19 days), II period (up to 33 days), and III period (up to 70 days), respectively, with the proportion daily growth rates of 3.0 ± 0.30, 15.2 ± 0.62 and 6.5 ± 0.25% g.day^–1^, respectively, being the values in the intermediate period significantly higher (Fig. 2).

### Environmental parameters

**Figure 3:**
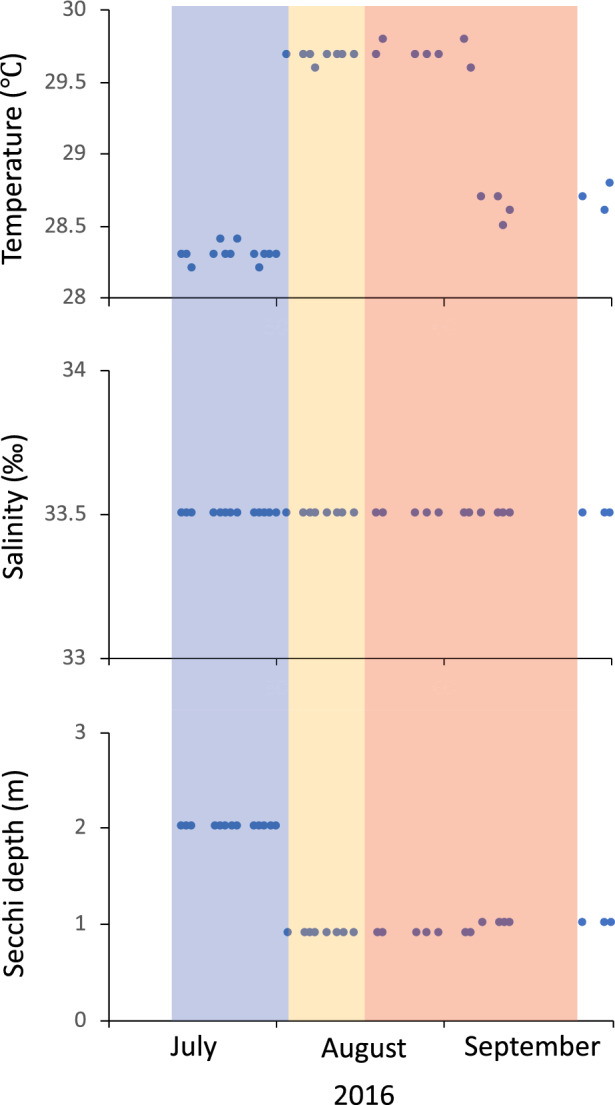
Variation of sea surface temperature, salinity, and turbidity (Secchi depth) in the cultivation zone of *Kappaphycus alvarezii* on the coast of the province of Santa Elena, Ecuador. The three colours highlight the corresponding three different sampled period months from July to September 2016.

**Figure 4:**
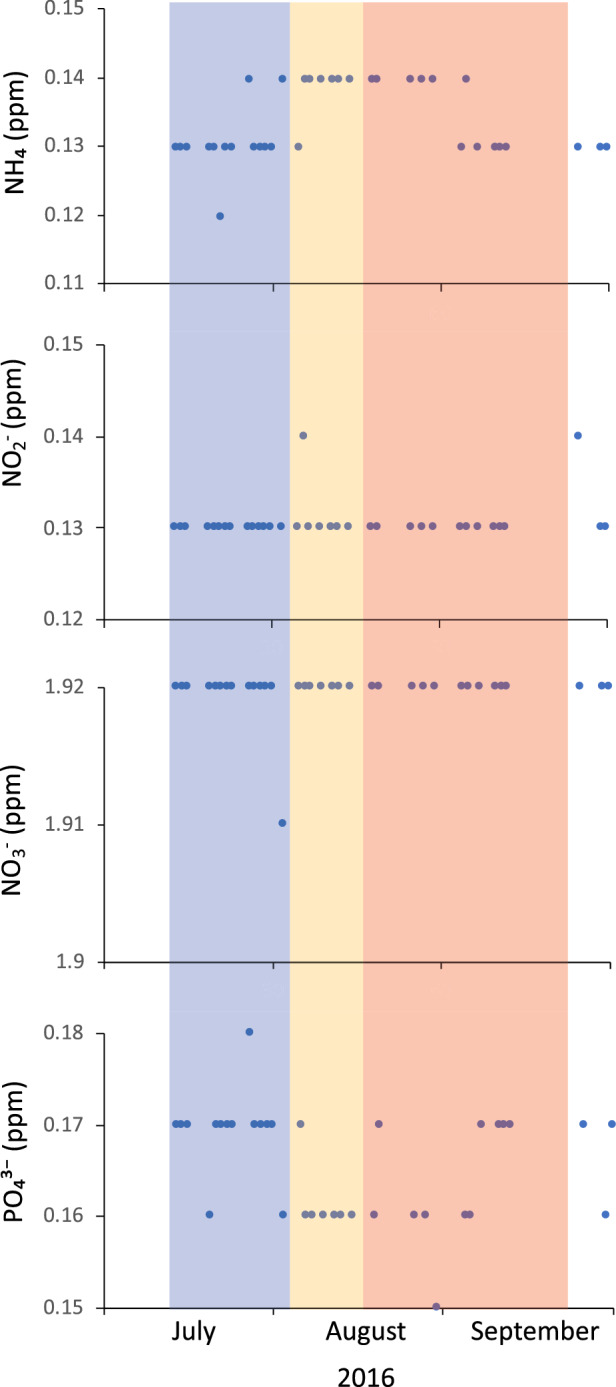
Variation in the concentration of ammonium, nitrite, nitrate, and phosphate, measured in the sea surface water of the cultivation zone of *Kappaphycus alvarezii* on the coast of the province of Santa Elena, Ecuador. The three colours highlight the corresponding three different sampled period months from July to September 2016.

The sea surface temperature presented an average of 28.9 ± 0.64 °C, with little difference among time periods (< 2 °C); however, the maximum temperatures recorded during the intermediate period (29.7 ± 0.06 °C) was significantly higher than the temperature in the final (28.7 ± 0.09 °C) and initial (28.3 ± 0.06 °C) periods (Fig. 3). The average turbidity was 1.3 ± 0.52 m, with a maximum of 2.0 ± 0.00 m in the initial period, significantly higher than that recorded in the intermediate (0.91 ± 0.025 m) and final period (1 ± 0.00 m). Regarding the dissolved inorganic nitrogenous compounds in the cultivation area, the values of NH_4_^+^, NO_2_^−^ and NO_3_^–^, were generally 0.13, 0.13 and 1.92 ppm, respectively, without showing significant differences among the study periods (Fig. 4). Phosphate showed greater variability among periods, with values between 0.16 and 0.18, being the values in the initial period significantly higher.

### Carrying capacity

Since each cultivation unit requires an area of 0.16 ha, the concession area in Bahía Las Conchas has a carrying capacity of the water body equivalent to 86.2%, which represents a total of 53 cultivation units in the concession system with horizontal floating rafts (Table 1).

**Table 1:** Carrying capacity of the concession area of Bahía Las Conchas in the Province of Santa Elena, Ecuador.

Parameters	Values
Area of concession	10 ha
Recommended area for the raft	0.16 ha
Capacity of the water body	86.2%
Carrying capacity	8.6 ha
Number of floating rafts	53

### Economic viability

The 10-year financial study revealed a payback time from the fourth year of project start-up, with a return of 19% on the expected total income, with an expected annual production of 121.3 T in dry weight of *K. alvarezii*. The financial viability of the cultivation of the macroalga in floating farming systems was determined by the net present value (NPV) of $58,326.63, the internal rate of return (IRR) of 16.46% and the benefit–cost ratio (B/C) (1.67) (Table 2).

**Table 2:** Financial evaluation of the floating raft culture of *Kappaphycus alvarezii* in the Pacific coast of Ecuador.

Economic variables and management	
Number of algae planted per year (11 production cycles)	1.244,122
Number of harvested algae per year (11 production cycles)	1.119,709.8
Cumulative survival*	90.00%
Average dry weight of harvested algae	108.3 g
Dry weight yield	1711.94 g/m^2^
Annual dry weight yield (11 production cycles)	121.3 T
Annual sales revenue	162,413.33 $
Annual seed cost	60,666.67 $
Annual labour costs^a^	55,200.00 $
Other annual production costs	29,797.60 $
Other administrative expenses	34,191.34 $
Total modules of rafts	53.00
Financial indicators of profitability	
Net present value (NPV)^b^	58,326.63 $
Share of net income in relation to revenues	19%
Internal rate of return (IRR)^b^	16.46%
Benefit–cost ratio (B/C)^b^	1.67
Capital payback period (years)	4.30

^a^A total of 259,200 man-hours are estimated per production cycle.

^b^A discount rate of 9.96% is used.

*Although no mortality was observed in the study, we conservatively estimate a mortality of 10%.

## Discussion

Our trial study provides relevant new data on the biological feasibility of elkhorn sea moss (*K. alvarezii*) cultivation on the coast of the province of Santa Elena, Ecuador. During cultivation, we did not observe notable losses or damage to algae culture due to rupture, nor damage by grazers, which would attribute large losses in biomass production^35^. The recorded biomass growth after 70 days of cultivation, was more than 9 times (wet and dry) the original biomass sown, obtaining an average weight of 1600 g (144 g dry biomass) per propagule. In this period the % DGR was 6.5 ± 0.25% g.day^–1^.

The growth was more accelerated in the exponential phase (from day 20 to 33), reaching biomasses of about 1150 g (108 g dry biomass). During period II (initial to 33 days) the % DGR was 15.2 ± 0.62% g.day^–1^, which was more than twice if the algae were harvested after 70 days and 5 times more if the seaweed was harvested after 20 days. Slow growth in the first 20 days and after day 33 is characteristic in the culture of this species^36^. This suggests that the cultured biomass should be harvested after 30–35 days from out planting, when the highest growth rates are found^37^. Harvest time depends on the product yield and quality, and certain authors report that at a time close to a month after sowing, when the extraction of algae products (e.g. carrageenan) is feasible and recommended^36,37^. While, it has been documented that *K. alvarezii* has a lower concentration of carrageenan when harvested in a shorter period, other studies^49–51^ have recommended harvesting over longer periods of time, about 45 days after sowing.

When comparing the % DGR of the present study harvesting *K. alvarezii* at 33 days, with previous studies in other tropical and subtropical regions (Table 3), the daily growth rate obtained for *K. alvarezii* growth (15.2% g.day^–1^) exceed all previous reports, either from studies carried out in the Atlantic (in Brazil, with the highest rate of 8.9% g.day^–1^) or in Asia with highest reported in India of 14% g.day^–1^, but in several other countries did not exceed 10.8% g.day^–1^. The high growth rate detected in the current study, in combination with environmental and littoral geography conditions of an extensive marine coastline (670 km) allow us to elucidate the positive biomass conditions for macroalgae production in Ecuador. Moreover, the possibility of culturing *K. alvarezii* in the upper estuary zone of Ecuador, where there is already a high level of aquaculture activity by coastal communities, reinforce the relevance of introducing the production of new species to diversify the aquaculture of the entire tropical Pacific region, in particular in Ecuador, where more than 95% is directly linked to shrimp farming^1^.

**Table 3:** Culture parameters with estimated daily growth rate in percentages of the main culture studies of *Kappaphycus alvarezii*.

Initial weight of propagules	Harvest time in days	(% day^–1^)	Equation of % DGR used	Location	References
150–500 g	120	3.5–5.6	[(InWf/InWo)/t] × 100	Hawaii	^38^
150 g	30	1.9–6.2	[(InWt–In Wo)/t] × 100	Hawaii	^11^
32–36 g	18	1.9–4.6	[(InWf/InWo) × 100]/t	China	^39^
100–150 g	60	3.7–7.2	[In (Wf–Wo)/t × 100	Philippines	^40^
2 kg por line 7 m	72	5.9–8.9	[(Wf–Wo/Wo)] × 100/t	Philippines	^10^
1.5 g	120	0.1–8.1	[(100 In (Wf/Wo)/t] - 1	Shikoku Japan	^41^
2 kg per line of 5 m	60	1.1–3.4	[In (Wf-Wo)/t] × 100	Philippines	^42^
400 g/m^2^	120	3.2–10.8	[(Wt/Wo)^1/t^–1] × 100	Vietnam	^7^
100–150 g	30	4.5–8.9	[(Wt/Wo)^1/t^–1] × 100	Brazil	^19^
2 kg per line of 5 m	90	2.3–4.2	[In (Wf–Wo)/t] × 100	Philippines	^43^
5 g	90	0.3–5.5	[(Wt–W0)/^1/t^] × 100	India	^44^
50 g	90	4.5–8.2	[(Wt/Wo)^1/t^–1] × 100	Brazil	^45^
~ 100 g	30	2.0–7.1	ln(Wt–Wo)/t × 100	Yucatán, México	^4^
20–50 g dry	30	2.5–6.6	[(Wt/Wo)^1/t^–1] × 100	Brazil	^46^
100 g	45	3.9–14.0	In (Wf–Wo)/t × 100	India	^47^
50 g	40	2.4–3.7	(lnWt/lnWo)/t × 100	Indonesia	^48^
20 g	40	2.6–4.3	[(InWf/InWo)/t] × 100	Indonesia	^36^
159.3 ± 12.74 g wet	19	3.0	[(Wf–Wo)^1/t^–1] × 100	Ecuador	This study
	33	15.2			
	70	6.5			

Wo initial weight (g), Wt final weight (g) t is the number of days of culture.

As such, to support the background idea of farming *K. alvarezii* and other macroalgae species in the region, more studies generating further evidence data should be promoted on the topic of assessing the production of macroalgae as one of the strategies to reduce the effects of climate change, counteract eutrophication and the crisis of biodiversity lost^52^. Given that macroalgae aquaculture is nowadays widely recognised as a strategic pathway to achieve a blue economy to meet more sustainability objectives^53,54^, the cultivation of macroalgae across the tropics, should be considered as one of main focus of public policies where the government, academic and private sector sectors must interact.

In our experimental design, although environmental factors related to the growth of macroalgae were recorded, there were no correlations detected with growth rates, except for the previously underlined intermediate II period, for which a positive correlation between increased temperature and higher biomass was observed. While temperature is a factor that modulates growth in aquatic organisms, including macroalgae and particularly photosynthesis^55^, the difference among the studied periods did not exceed 2 °C, which was possibly not decisive in causing notable physiological effects in the macroalgae. This suggests that differences in growth rate might be associated with endogenous factors of post-adaptability of the algae, after its initial growth phase^56^. However, an important feature of the higher % DGR found in *K. alvarezii* cultures in Vietnam, is that the temperature was notably higher (33 °C) than the one recorded in the present study, which reflects the higher metabolism at high temperatures of this species. Further studies are necessary to understand the effect of temperature in tropical ranges on this algae species. Though the species can grow at lower temperatures, such as those occurring in the subtropics (17–31 °C), its production is lower (e.g. South Japan^41^ and Bahía de Ubatuba in Brazil^45,46^) than those observed in tropical areas, where the temperature is much higher and less variable^4,14,26,36,57,58^.

Regarding salinity measurements in our study, the culture in the province of Santa Elena was developed in a range of 30–35 ‰, which is considered optimal for *K. alvarezii*^8,9^. The chemical nutrients dissolved in the water measured as NH_4_^+^ and PO_4_^3–^, were higher than the minimum required values of 0.3 to 0.6 ppm, both in NH_4_^+^, NO_2_^–^ and NO_3_^–^^11–14^, and (PO_4_^3–^) 0.009–0.05 ppm, previously reported for a good development of the macroalgae^59^, which contributed to the good performance of the algae under cultivation. Both, the sowing density, and the depth of the cultivation set up, are also determining factors in productivity, and must be considered as one of the main factors affecting farm productivity^49,50^.

Quantification of the carrying capacity of an aquaculture system is important because its scale will determine the impact on the hydrodynamics of the area, the risk of spread of pests and diseases, as well as the probability of eutrophication due to the decomposition of the biomass^60^. However, eutrophication is an unlikely factor from the cultivation of this species as the biomass that breaks off and washes up on the shore is usually harvested due to its high commercial value. The calculations of the carrying capacity was based on multiple models and was adjusted to specific characteristics of the species and site where the project was performed^61,62^, the little information concerning to the cultivation of seaweeds is a limiting factor when comparing our results. For example, a recent work by Gomes Da Silva et al.^63^ showed that a plant cover area of 2 ha is considered by the Brazilian Government to have a low ecological impact due to the oceanographic characteristics in the Southeast region of that country. Being the area of this study less than the determined cultivation capacity (8.6 ha) for Santa Elena, and considering a maximum load capacity of 86.15%, would allow a production of 121.3 T in dry weight of algae for the total concession area of 10 ha.

Until now, the economic viability for this species has mainly been reported for “family scale” developments < 0.5 ha. For example, in Colombia where the internal rate of return (IRR) was 65%^22^, and in Brazil between 38.1 and 87.8%^64^. However, Nogueira and Henriques^64^, concluded that the financial unfeasibility for large-scale macroalgae production in Brazil, is because of the required plant cover area and current legislation. New economic models in multitrophic cultures of *Kappaphycus* algae with bivalves^63^ suggest features like those here reported, showing financial feasibility with an annual production of 121.3 T in dry weight of *K. alvarezii*, a period of recovery of 4.3 years of the investment and a rate of return of 16.46%. This activity generates an important socio-economic contribution to the sector (mainly constituted of associations of artisanal fishermen) since it guarantees the use of 259,200 man-hours for its development. Unlike Nogueira & Henriques^64^, this study demonstrates that in Ecuador the cultivation of macroalgae on a large scale is possible, based on financial viability, carrying capacity of the site and current legislation.

To our knowledge, this is the first report on the cultivation of *K. alvarezii* and its feasibility in the Tropical Eastern Pacific waters. It was observed that the algae increased its biomass by more than 7 times after 33 days of cultivation, with an average daily growth rate of 15.2% g.day^–1^. These values are almost three times higher than those proposed as suitable for commercial cultivation of eucheumatoides seaweeds worldwide^16^. The productivity and growth rates show the biological feasibility of *K. alvarezii* cultivation in the province of Santa Elena under the previously described conditions of temperature, salinity, and nutrients.

The load capacity established in the study area was 53 floating rafts in total, with a profitability of 67%. Although this profitability seems to be low, it is higher than that established by Gomes Da Silva et al.^63^ in multitrophic culture (*K. alvarezii* with *Perna perna*, and *Nodipecten nodosus*) carried out in the state of Santa Catarina, Brazil. The data supplied for the economic feasibility exercise is real and comes from government support for the “Mariculture Macroproject on the Ecuadorian Coast”, which is subject to various administrative control procedures that normally slow down and increase the cost of the initial investment, so the projection of costs in the present study could be overestimated.

We recommend continuing with the evaluation and refinement of *K. alvarezii* cultivation practices in the same study region and similar areas along the coast of Ecuador. Studies should include optimization of mass, number and seeding distance of propagules, control of biofouling, improvement of the product, etc. together with more detailed studies on phytopathology, product quality and commercialization. Special emphasis should be given to social inclusion, particularly in the active participation of women in the cultivation. The effect of different environmental factors on the culture should be evaluated before managing a large-scale commercialization phase, based on a *K. alvarezii* mariculture establishment with an adequate social and environmental impact within the framework of productive sustainability. Finally, given the systemic services that macroalgae can generate, we encourage and recommend to focus efforts on carbon sink studies for *K. alvarezii* cultures, as well as evaluating the effect on eutrophication reduction from discharge water systems from the shrimp industry and other similar discharge systems.

## Data Availability

Data supporting the conclusions of this study are available from the corresponding author upon request.
